# Comparison of Seven Chemical Pretreatments of Corn Straw for Improving Methane Yield by Anaerobic Digestion

**DOI:** 10.1371/journal.pone.0093801

**Published:** 2014-04-02

**Authors:** Zilin Song, GaiheYang, Xiaofeng Liu, Zhiying Yan, Yuexiang Yuan, Yinzhang Liao

**Affiliations:** 1 Chengdu Institute of Biology, Chinese Academy of Science, Chengdu, Sichuan, PR China; 2 Research Center of Recycle Agricultural Engineering Technology of Shaanxi Province, Northwest A&F University, Yangling, Shaanxi, PR China; Oak Ridge National Laboratory, United States of America

## Abstract

Agriculture straw is considered a renewable resource that has the potential to contribute greatly to bioenergy supplies. Chemical pretreatment prior to anaerobic digestion can increase the anaerobic digestibility of agriculture straw. The present study investigated the effects of seven chemical pretreatments on the composition and methane yield of corn straw to assess their effectiveness of digestibility. Four acid reagents (H_2_SO_4_, HCl, H_2_O_2_, and CH_3_COOH) at concentrations of 1%, 2%, 3%, and 4% (w/w) and three alkaline reagents (NaOH, Ca(OH)_2_, and NH_3_·H_2_O) at concentrations of 4%, 6%, 8%, and 10% (w/w) were used for the pretreatments. All pretreatments were effective in the biodegradation of the lignocellulosic straw structure. The straw, pretreated with 3% H_2_O_2_ and 8% Ca(OH)_2_, acquired the highest methane yield of 216.7 and 206.6 mL CH_4_ g VS ^−1^ in the acid and alkaline pretreatments, which are 115.4% and 105.3% greater than the untreated straw. H_2_O_2_ and Ca(OH)_2_ can be considered as the most favorable pretreatment methods for improving the methane yield of straw because of their effectiveness and low cost.

## Introduction

Biomass is considered as a valuable alternative energy source to fossil fuels worldwide because it can be converted into various available forms of energy, such as heat, electricity, steam, biogas, hydrogen, and liquid transportation biofuels [1], [2]. As the largest agricultural country in the world, China has an abundance of biomass resources. Approximately 800 million tons of various crop residues are produced in China per year, of which corn and wheat straw account for 216 and 135 million tons, respectively [3]. Crop straws have not been widely used for bioenergy production because of the undeveloped conversion technology. Instead, many crop straws are burnt or directly dumped into the fields, causing serious environmental pollution and degraded soil conditions [4]. Therefore, the development of inexpensive and effective technologies for corn straw utilization is necessary.

Anaerobic digestion (AD) of agricultural straw for bioenergy production is widely used as a promising and alternative energy source to fossil fuels [5]. This technology has been considered as the main commercially viable option for the both treatment and recycling of biomass wastes, and thus is of great interest from an environmental and bioenergy source perspective [6]. However, the efficiency of this technology in treating agricultural straws is limited because the components of straw (lignin, cellulose, and hemicellulose) are difficult to degrade; thus, soluble compounds with low molecular weights are less available for anaerobic microorganisms [7]. Straw pretreatments prior to AD is a simple and effective method of improving the biodegradability of lignocellulosic materials because it can decompose cellulose and hemicellulose into relatively readily biodegradable components while breaking down the linkage between polysaccharide and lignin to make cellulose and hemicellulose more accessible to bacteria [8], [9].

Pretreatment methods mainly include physical methods [2], [10], chemical methods [11]–[14], biological methods [1], [15], and a combination of the abovementioned methods [16], [17]. Compared with physical and biological treatment methods, chemical pretreatment methods are predominantly used because they are inexpensive and are effective for enhancing the biodegradation of complex materials [18]. In chemical pretreatment methods, sulphuric acid (H_2_SO_4_), hydrochloric acid (HCl), hydrogen peroxide (H_2_O_2_), acetic acid (CH_3_COOH), sodium hydroxide (NaOH), lime (Ca(OH)_2_), and aqueous ammonia (NH_3_.H_2_O) are the common chemicals to improve AD performance of agricultural residues [19]–[26]. For instance, Fernández-Cegrí et al. [2] reported that the methane yield of sunflower oil cake with Ca(OH)_2_ is 130 CH_4_ g^−1^ COD, which is 25% higher that of the untreated sample. Zhu et al. [12] found that NaOH-pretreated corn stover yields 37.0% to 72.9% higher biogas productions than the untreated sample. Kang et al. [23] showed that the optimal conditions for the ethanol production of rapeseed straw is through immersion in aqueous ammonia containing 19.8% ammonia water at 69.0°C for 14.2 h. In addition, H_2_SO_4_, HCl, and CH_3_COOH pretreatments have been used to improve the AD of lignocellulosic materials [24], [25]. However, the most economically and effectively favorable treatments, among these, have yet to be identified. Additionally, the optimal concentration for the favorable pretreatment has been scarcely reported. Such information is important for the reasonable and efficient utilization of agricultural residues. The present study compared the effects of four acid and three alkaline pretreatments on the lignocellulosic compositions and methane yield of corn straws by AD. Our objective was to determine the most cost-effective pretreatment methods for enhancing the methane yield of straws.

## Materials and Methods

### Raw Material

Corn straw was obtained from a local villager near the Northwest A&F University (Yangling, Shaanxi, China). Prior to use, the straws were air dried, cut into lengths of 20 mm to 30 mm using a grinder, and then individually homogenized for further use. The full composition and main features of the corn straw were as follows (mean values of three determinations ± standard deviations): total solids (TS), 93.6%±2.8%; volatile solids (VS), 86.7%±1.9%; total carbon (TC), 42.3%±2.8%; total nitrogen (TN), 0.82%±0.05%; hemicellulose, 28.8%±1.4%; cellulose, 49.3%±1.8%; and lignin, 7.5%±0.4%.

### Pretreatment Process

Seven pretreatment methods were used in this study, including four acid treatments (H_2_SO_4_, HCl, CH_3_COOH, and H_2_O_2_) and three alkaline treatments (NaOH, Ca(OH)_2_, and NH_3_·H_2_O). The reagents were purchased from Sinophram Chemical Reagent Co. Ltd, Beijing, China. The chosen pretreatment conditions were based on previous studies [9], [20] and carried out using different concentrations of reagents. Acid reagents (H_2_SO_4_, HCl, H_2_O_2_, and CH_3_COOH) at concentrations of 1%, 2%, 3%, and 4% (w/w) and alkaline reagents (NaOH, Ca(OH)_2_, and NH_3_·H_2_O) at concentrations of 4%, 6%, 8%, and 10% (w/w) were used for the pretreatments. The corn straw not pretreated with any chemicals was used as the control. Each pretreatment was conducted in triplicate.

Dried corn straw (500 g) was soaked in the prepared 1.5 L solutions contained in beakers, yielding straw samples with 75% moisture. All prepared beakers were covered with plastic films, secured with a plastic ring, and then stored in a chamber at an ambient temperature of 25±2°C for 7 days. After the pretreatment, the straws were removed from the beakers, dried in an electronic oven (HengFeng SFG-02.600, Huangshi, China) at 80°C for 48 h, and then kept in a refrigerator for composition determination and AD experiments to investigate the effect of different chemical treatments on methane yield.

### Anaerobic Digestion

The digestion experiment was conducted according to methods described by Song et al. [22] using laboratory-scale simulated anaerobic digesters in 1 L Erlenmeyer flasks. The batch reactors were used to determine the digestion levels of the straws with different pretreatments. Each pretreated straw was used as the digestion material, with the untreated straw as the control. The digestion inoculum was collected from an anaerobic digester in a model village powered by household biogas (Yangling, Shaanxi, China). This particular inoculum was selected because of its high methanogenic activity. The characteristics and features of the anaerobic inoculum used were as follows: pH, 7.6±0.1; TS, 86.6%; and VS, 47.5%. The digestion material (500 g) and inoculums (200 g) were added to each digester, followed by deionized water to obtain an 8% TS content. They were stirred and placed in a thermostatic water bath at the mesophilic condition of 37±1°C for 35 d of AD. All reactors were tightly sealed with rubber septa and screw caps. All reactors were gently mixed manually at approximately 1 min d^−1^ prior to biogas volume measurement to ensure mixing of the reactor contents. Moreover, 200 g of the inoculums was digested to serve as the blank in determining the normalized methane yield of the inoculum by itself. The digestion of each pretreatment was performed in triplicate.

### Analysis and Calculations

The volume of biogas was measured by water displacement. The methane content in the produced biogas was analyzed with a fast methane analyzer (Model DLGA-1000, Infrared Analyzer, Dafang, Beijing, China). The TS, VS, TN, and pH of the materials were measured according to the *Standard Methods for the Examination of Water and Wastewater* of the American Public Health Association [27]. The pH was tested once every 5 d. TC content was analyzed using the method described by Cuetos et al. [28]. The C/N ratio was determined by dividing the total organic carbon content to the TN content. The volatile fatty acid (VFA) was analyzed using a colorimetric method [29], and the result was expressed in terms of acetic acid content. The cellulose, hemicellulose, and lignin contents were analyzed based on the methods previously described by Wang and Xu [30].

### Data Analysis

Data is expressed as mean ± standard deviation (SD) of the triplicate measurements. Differences between mean values were examined by ANOVA. Comparisons among means were made using the Duncan multiple range test, and significance was set at *P*<0.05. All statistical analyses were performed using the software program SPSS 15.0. (SPSS Inc., Chicago, USA).

## Results and Discussion

### Effects of Pretreatments on the Chemical Composition of Corn Straw

The aim of the pretreatments was to change the raw material properties, remove or dissolve lignin and hemicellulose, and reduce the crystallinity of cellulose [31]. In the present study, both acid and alkaline pretreatments changed the lignocellulosic composition of corn straw (Tables 1 and 2). Compared with the untreated straw, the hemicellulose and cellulose contents of the acid-treated straw significantly decreased by 6.6% to 66.0%, and 4.4% to 54.3% (*P*<0.05), and the hemicellulose and lignin contents of alkaline-treated corn straw decreased by 10.7% to 46.7%, and 10.8% to 60.7%. These results indicated that pretreatments are more effective in breaking down the lignocellulose matrix and in changing the chemical components of straw. Considerable amounts of lignocellulose appeared to be decomposed and converted into other soluble components that are available to anaerobic microorganisms [32].

**Table 1:** Effect of acid pretreatment on the chemical composition of corn straw.

Pretreatment	Concentration	Cellulose %	Hemicellulose %	Lignin %	TC %	C/N
H_2_SO_4_	1%	47.1±2.5 a	26.9±1.2 a	7.5±0.5 a	37.3±2.0 b	45.5±2.0 b
	2%	41.3±1.8 b	22.5±1.8 b	7.3±0.4 a	30.6±1.9 c	38.7±1.6 c
	3%	38.0±1.6 bc	16.2±1.2 c	7.3±0.4 a	30.3±2.1 c	37.9±1.1 c
	4%	36.1±1.6 c	13.0±0.9 d	6.7±0.5 a	25.3±1.5 d	30.9±3.2 d
Untreated		49.3±1.8 a	28.8±1.4 a	7.5±0.4 a	42.3±2.8 a	51.4±3.6 a
HCl	1%	46.7±2.2 a	26.2±1.9 a	7.9±0.5 a	37.1±2.0 b	44.2±1.8 b
	2%	40.4±2.0 b	22.2±2.0 b	7.2±0.7 a	32.4±2.0 c	39.5±0.9 c
	3%	38.2±1.6 b	17.3±1.0 c	6.4±0.6 a	29.2±1.9 c	38.4±2.1 c
	4%	35.4±0.8 c	14.5±1.3 d	6.9±1.0 a	26.1±1.2 d	32.6±0.7 d
Untreated		49.3±1.8 a	28.8±1.4 a	7.5±0.4 a	42.3±2.8 a	51.4±3.6 a
CH_3_COOH	1%	43.8±1.9 b	26.8±2.6 a	7.1±0.9 a	38.6±2.9 a	47.7±1.6 ab
	2%	37.4±2.4 c	21.7±1.1 b	6.7±0.5 a	34.8±0.9 b	46.4±1.9 b
	3%	34.2±0.9 d	18.1±1.4 c	6.8±0.5 a	29.5±0.9 c	36.9±2.6 c
	4%	30.4±1.5 e	15.1±0.5 d	6.7±0.7 a	26.4±1.6 d	32.2±0.7 d
Untreated		49.3±1.8 a	28.8±1.4 a	7.5±0.4 a	42.3±2.8 a	51.4±3.6 a
H_2_O_2_	1%	40.5±1.5 b	25.0±1.4 b	7.0±0.2 a	34.4±2.6 b	44.7±2.3 b
	2%	34.6±2.1 c	20.8±2.3 c	6.5±0.3 b	28.7±0.8 c	37.3±1.1 c
	3%	30.8±0.8 d	14.3±1.2 d	5.7±0.4 c	25.1±1.2 d	30.6±2.4 d
	4%	22.5±0.6 e	9.5±0.7 e	5.1±0.2 d	20.4±1.3 e	25.2±2.1 e
Untreated		49.3±1.8 a	28.8±1.4 a	7.5±0.4 a	42.3±2.8 a	51.4±3.6 a

Data are expressed as mean ± deviation of triplicate measurements. TC: Total carbon.

The ANOVA test was conducted to determine the differences between each pretreatment. Values with the same letters in each pretreatment indicate no significant difference at *P*<0.05.

**Table 2:** Effect of alkaline pretreatment on the chemical composition of corn straw.

Pretreatment	Concentration	Cellulose %	Hemicellulose %	Lignin %	TC %	C/N
NaOH	4%	48.0±3.9 a	23.8±1.4 b	6.7±0.5 a	39.3±0.8 a	49.1±1.0 a
	6%	46.1±3.0 a	20.6±0.9 c	5.5±0.5 b	35.4±2.3 b	46.0±0.8 b
	8%	46.7±2.2 a	16.2±0.9 d	4.6±0.3 c	33.7±1.6 b	42.1±2.1 c
	10%	47.4±2.6 a	11.3±1.2 e	4.0±0.2 d	28.1±1.2 c	34.7±1.3 d
Untreated		49.3±1.8 a	28.8±1.4 a	7.5±0.4 a	42.3±2.8 a	51.4±3.6 a
Ca(OH)_2_	4%	47.5±1.8 a	24.6±2.2 b	6.8±0.2 a	37.8±1.5 a	45.0±1.8 b
	6%	46.1±2.4 a	21.2±1.4 c	6.0±0.3 b	32.8±3.1 b	40.0±2.0 c
	8%	46.3±1.9 a	16.4±1.1 d	5.4±0.2 c	29.4±2.0 b	38.7±0.7 c
	10%	48.0±1.1 a	12.3±1.2 e	4.6±0.3 d	22.6±1.8 c	28.3±1.9 d
Untreated		49.3±1.8 a	28.8±1.4 a	7.5±0.4 a	42.3±2.8 a	51.4±3.6 a
NH_3_•H_2_O	4%	48.1±1.2 a	25.7±1.9 a	7.0±0.6 ab	39.2±1.9 a	48.4±2.1 a
	6%	45.4±3.3 a	22.4±0.8 b	6.6±0.3 b	36.6±2.5 b	48.8±0.4 a
	8%	45.9±3.0 a	18.6±1.8 c	6.2±0.2 c	33.2±0.9 b	41.5±1.8 b
	10%	45.1±2.9 a	17.8±1.1 c	5.5±0.2 d	30.7±1.6 c	37.4±1.8 b
Untreated		49.3±1.8 a	28.8±1.4 a	7.5±0.4 a	42.3±2.8 a	51.4±3.6 a

Data are expressed as mean ± deviation of triplicate measurements. TC: Total carbon.

The ANOVA test was conducted to determine the differences between each pretreatment. Values with the same letters in each pretreatment indicate no significant difference at *P*<0.05.

Guo et al. [20] reported that corn stalk mainly lost its hemicellulose and cellulose fractions after the acid treatment and lost its lignin fraction after the alkaline treatment. Fernández-Cegrí et al. [2] observed that H_2_SO_4_ cannot dissolve the lignin of sunflower oil cake, maintaining the same proportion as that of the untreated case. They also found that alkali pretreatments give higher removal levels of lignin compared with other reagents regardless of the temperature effect. The present study revealed a similar phenomenon that acid and alkaline pretreatments had different effects on the lignocellulose composition. In the case of acid reagents, hemicellulose and cellulose contents significantly decreased while the lignin content remained constant in the treated and untreated samples, except when the H_2_O_2_ was used that the lignin content decreased by 6.7% to 32.0%. The alkaline treatment was mainly effective in removing the lignin fraction. The effectiveness of degrading the lignocellulosic structure usually depends on the type of pretreatment method used, because of the attack on the different parts of the substrate by different chemicals. Acid pretreatment results in disruption of covalent bonds, hydrogen bonds, and Van der Waals forces that hold together the biomass components, which consequently causes the solubilization of hemicellulose and the reduction of cellulose [33]. In contrast, alkali treatment breaks the links between lignin monomers or between lignin and polysaccharides that makes the lignocelluloses swell through saponification reactions [34]. Among the pretreatments, H_2_O_2_ and NaOH showed the highest solubilization of hemicellulose cellulose, and lignin contents. This trend can be attributed to the strong oxidation ability of H_2_O_2_ [35] and the high alkalinity of NaOH that allow them to break down the lignocellulose matrix to change the chemical components of the straw. The increased degradation of lignocellulosic materials by H_2_O_2_ and NaOH suggests that these two chemicals are the most effective in degrading the lignocellulosic structure of corn straw.

The C/N ratio of anaerobic feedstock is significant for AD performance [36]. Analysis of the C/N ratio showed that the percentage of C in the pretreated straw significantly decreased with increasing chemical concentration (*P*<0.05, Tables 1 and 2). The decrease in TC content also affirmed this result. Although the C/N ratio in the pretreated straw was lower than that of the untreated sample, it was still higher than the optimum C/N ratio of feedstock materials (between 20 and 30) [36]. Therefore, the pretreated straw still represents a good co-digestion biomass because it provides a higher carbon fraction for digestion.

### Effects of Pretreatments on the Methane Yield of Corn Straw

**Figure 1:**
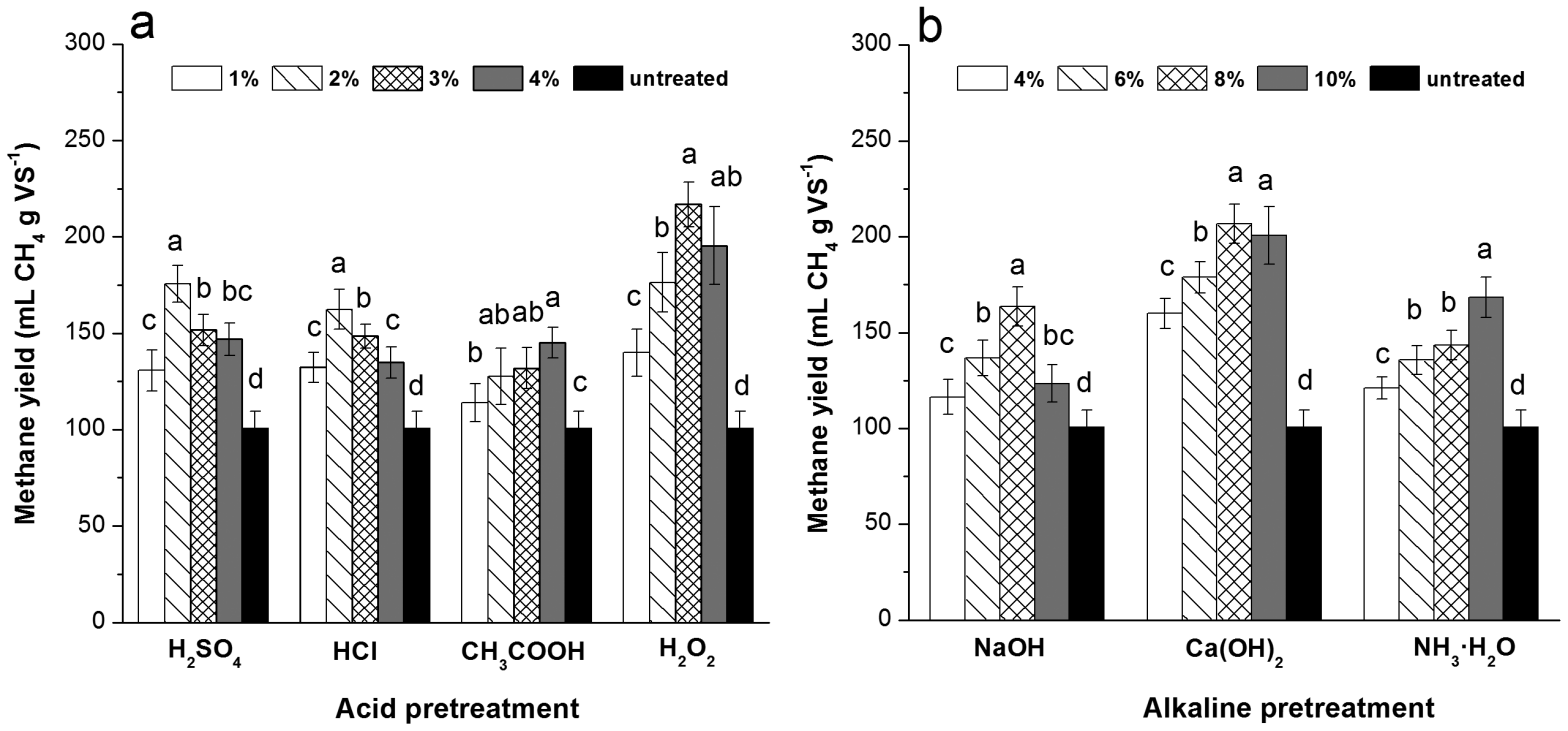
Effect of pretreatments on the methane yield of corn straw. (a) Acid pretreatment; (b) Alkaline pretreatment. Data was expressed at mean ± deviation of triplicate measurements. The ANOVA test was conducted to determine the differences between each pretreatment. Values with the same letters in each pretreatment indicate no significant difference at *P*<0.05.

The methane yield, defined as CH_4_ production per unit volatile solids (in mL CH_4_ g VS^−1^), was determined to compare the energy conversion efficiency and the improvement in biodegradability (Fig. 1). As shown in Fig. 1, the straws pretreated by acid and alkaline had significantly increased methane yields (*P*<0.05), i.e., an approximate 10.3% to 115.4% higher yield than for the untreated samples. These results are consistent with previous studies [11], [17] which verified the effectiveness of chemical pretreatment in improving biodegradability and enhancing bioenergy production. This phenomenon can be explained by the fact that alkaline and acid pretreatments promote organic solubilization and increase the surface area available for enzymatic action [31]. Chemical pretreatments have different effects on the anaerobic digestibility of corn straw. The methane yield was not improved as the chemical concentration increased. The highest methane yield was achieved at different concentrations for the seven pretreatments. For instance, the highest methane yield was achieved by H_2_SO_4_ and HCl at 2% concentration, CH_3_COOOH at 4%, H_2_O_2_ at 3%, Ca(OH)_2_ and NaOH at 8%, and NH_3_·H_2_O at 10%. The reason may due to the fact that successful biogasification is not only affected by the sufficient soluble component available but also by anaerobic bacteria. More soluble components from the biodegradation of the lignocellulosic composition need more bacterial to assimilate them. In the present study, the same amount of inoculums (200g) was applied in each digestion experiment, thus, the relative shortage of inoculums could be responsible for the lower methane yield of the chemical pretreatment with high concentration. Among the acid and alkaline treatments, H_2_O_2_ and Ca(OH)_2_ respectively produced the highest methane yield in the straw. This result suggests that H_2_O_2_ and Ca(OH)_2_ are best for improving the methane yield of corn straws compared with the other pretreatments. The methane yield was significantly heightened as the H_2_O_2_ concentration increased from 1% to 3% and 4%. However, the methane yield did not increase with further dose increases, showing no significant difference between 3% and 4%. The same trend was also observed for the Ca(OH)_2_ pretreatment at concentrations between 8% and 10%. The presence of excessive H^+^ in 4% H_2_O_2_ and OH^−^ in the 10% Ca(OH)_2_ pretreatment can cause toxicity to the methanogens thereby inhibiting their activity and interfering with their metabolism [37]. Therefore, 3% and 8% are the most suitable concentrations for the H_2_O_2_ and Ca(OH)_2_ pretreatments of corn straw, respectively.

### Effects of Pretreatments on VS Reduction of Corn Straw

**Figure 2:**
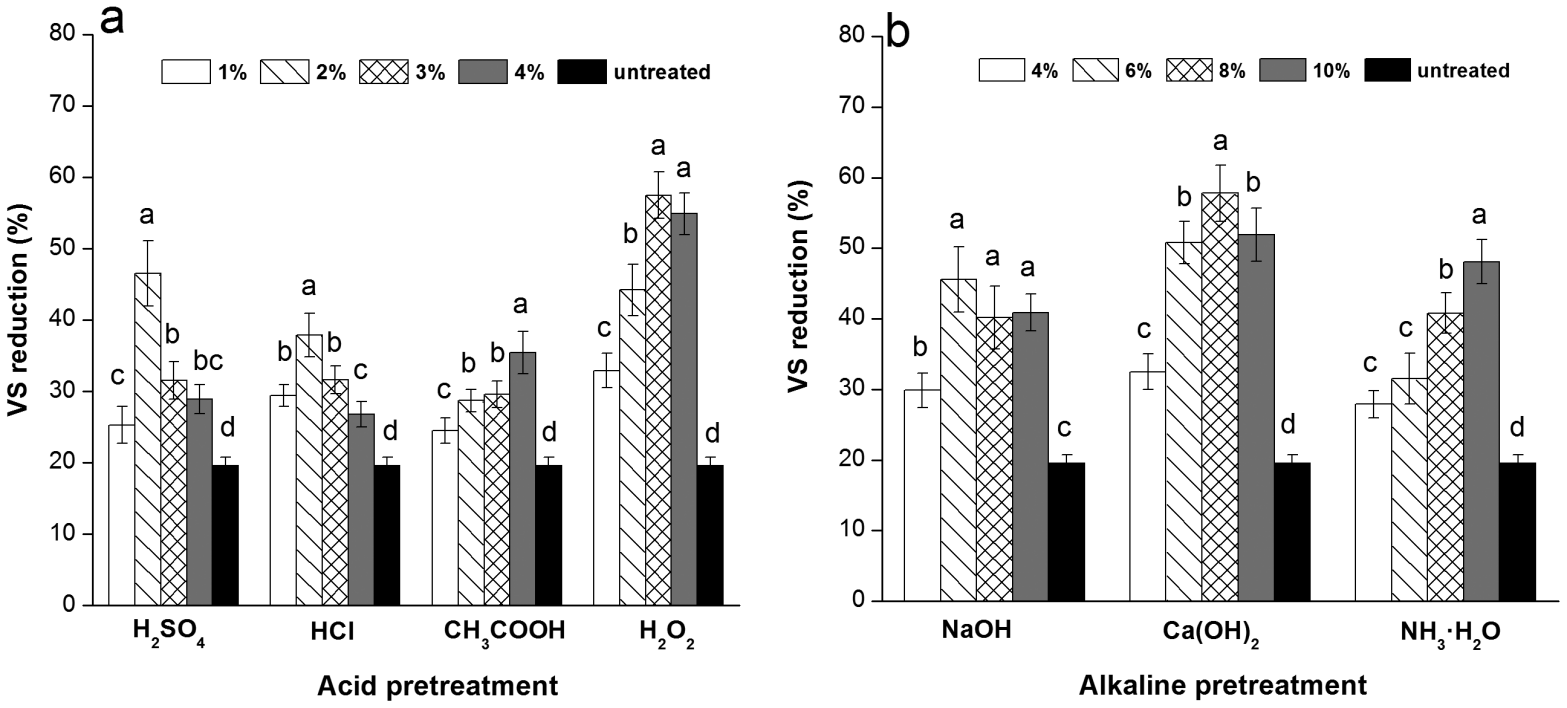
Effect of pretreatments on the VS consumption of corn straw. (a) Acid pretreatment; (b) Alkaline pretreatment. Data was expressed at mean ± deviation of triplicate measurements. The ANOVA test was conducted to determine the differences between each pretreatment. Values with the same letters in each pretreatment indicate no significant difference at *P*<0.05.

Methane is generated from the conversion of substrates; thus, the methane yield can be determined by reductions in the amount of dry matter of the substrate, as represented by VS. The VS reductions in the straw are shown in Fig. 2. Consistent with previous studies [22], the chemically-treated corn straw obtained higher VS reductions than untreated samples and exhibited reduction of 57.3% to 70.0% for the acid pretreatment and 57.5% to 70.8% for the alkaline pretreatment. 3% H_2_O_2_ and 8% Ca(OH)_2_ yielded the greatest reduction in the amount of dry matter of the substrate. The pretreatment triggers the conversion of VS into soluble compounds, including sugar, starch, pectin, tannin, cyclitol, and some inorganics, which become available to anaerobic microorganisms. Generally, this treatment contributes to a substantial improvement in the biodegradability of corn straw. High methane production requires more substrates for digestion; thus, increased VS reductions could explain why the methane yield of the treated straw was highly improved.

### Effects of Pretreatments on pH during AD

To investigate the effect of pretreatment on the VFA and pH during the AD of corn straw, the optimal concentration of each pretreatment for methane production was selected as follows: 2% H_2_SO_4_, 2% HCl, 4% CH_3_COOH, 3% H_2_O_2_, 8% NaOH, 8% Ca(OH)_2_, and 10% NH_3_·H_2_O.

**Figure 3:**
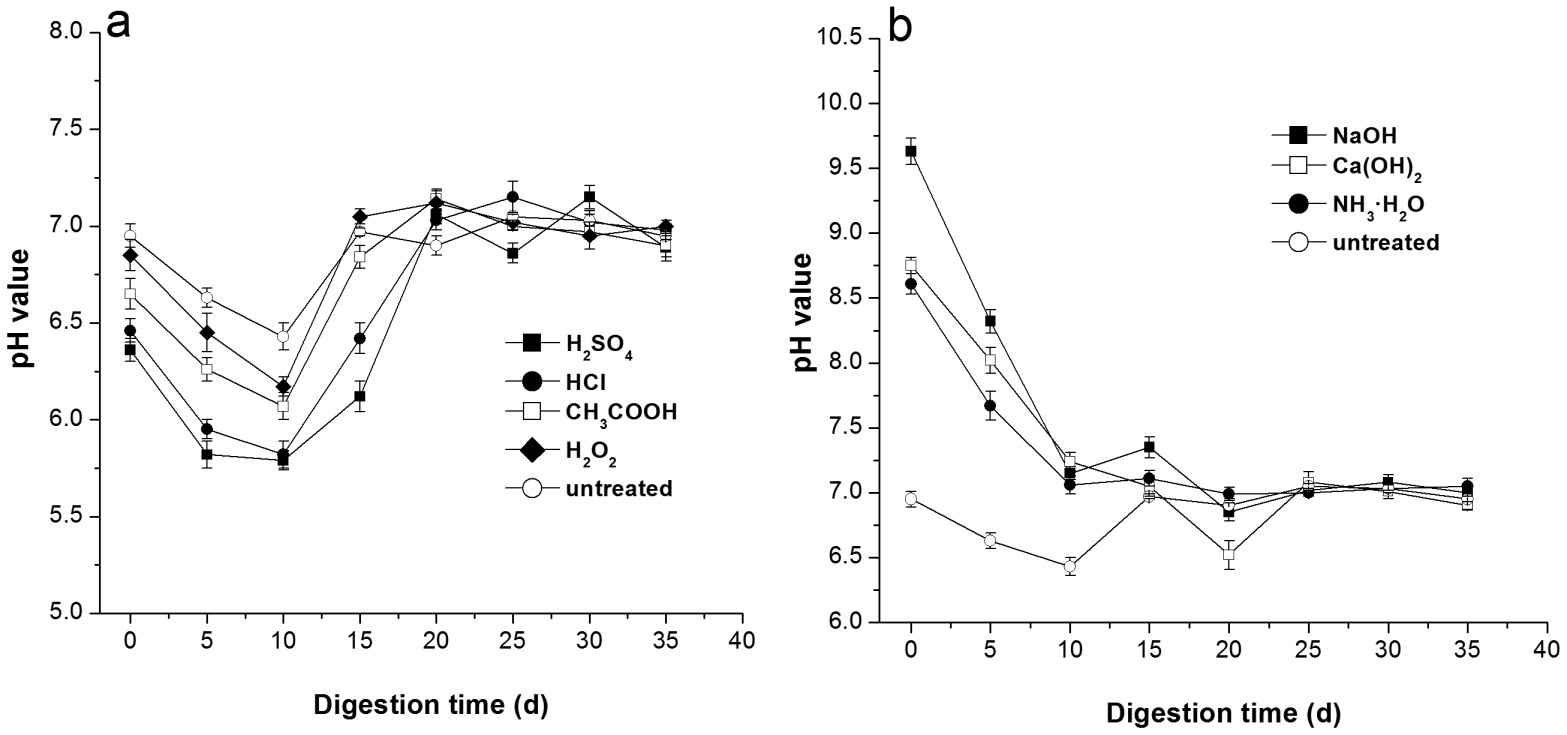
Change in the pH of pretreated corn straw during digestion. (a) Acid pretreatment; (b) Alkaline pretreatment. Data was expressed at mean ± deviation of triplicate measurements.

Fermentative microorganisms can function in a wider pH range of between 4.0 and 8.5 [38]. In the present study over the first 10 d, the pH of the fermentation broth of the acid-pretreated corn straws was below 7.0 (Fig. 3), whereas that of the three alkaline-pretreated corn straws was over 7.0. The pH curves of all pretreatments were similar, showing a decreasing trend in the initial 10 d and an increasing trend thereafter, slight fluctuations between days 10 to 20. At the end of the fermentation, all pretreatments maintained a pH of approximately 7.0. This trend can be attributed to the variation in VFA concentration because the production of VFA during AD decreases pH. The highly concentrated substrate at the initial phase of AD supplies sufficient organic acid from the degradation of hemicellulose, cellulose, lignin, and VS for the methanogens [20], which decreases pH and accelerates methanogen growth. As digestion proceeded, the content of organic acid gradually decreased with the consumption by the methanogens, which increased the pH. The shortage in organic acid limited the activities of the methanogens but stimulated the acidogens, which increased the amount of organic acids and the dropped the pH. The activity of the methanogens increased again when the organic acid accumulated to an extent, which increased the pH. However, compared with the dramatic fluctuation in the initial phase of AD, the change in the pH in the middle–late phase was slightly heightened because the concentration of the organic acid in the substrate was not as high as the initial concentration. The lack of significant differences in the pH for all pretreatments at the end of AD indicates that these pretreatments can recover the pH. As shown in Fig. 3, the pH of the fermentation broth of the pretreated corn straw markedly declined compared with that of the untreated corn straw. This result can be ascribed to the various acids in the soluble substance of the pretreated straw being significantly higher than that of the untreated straw.

### Effects of Pretreatments on VFA during AD

**Figure 4:**
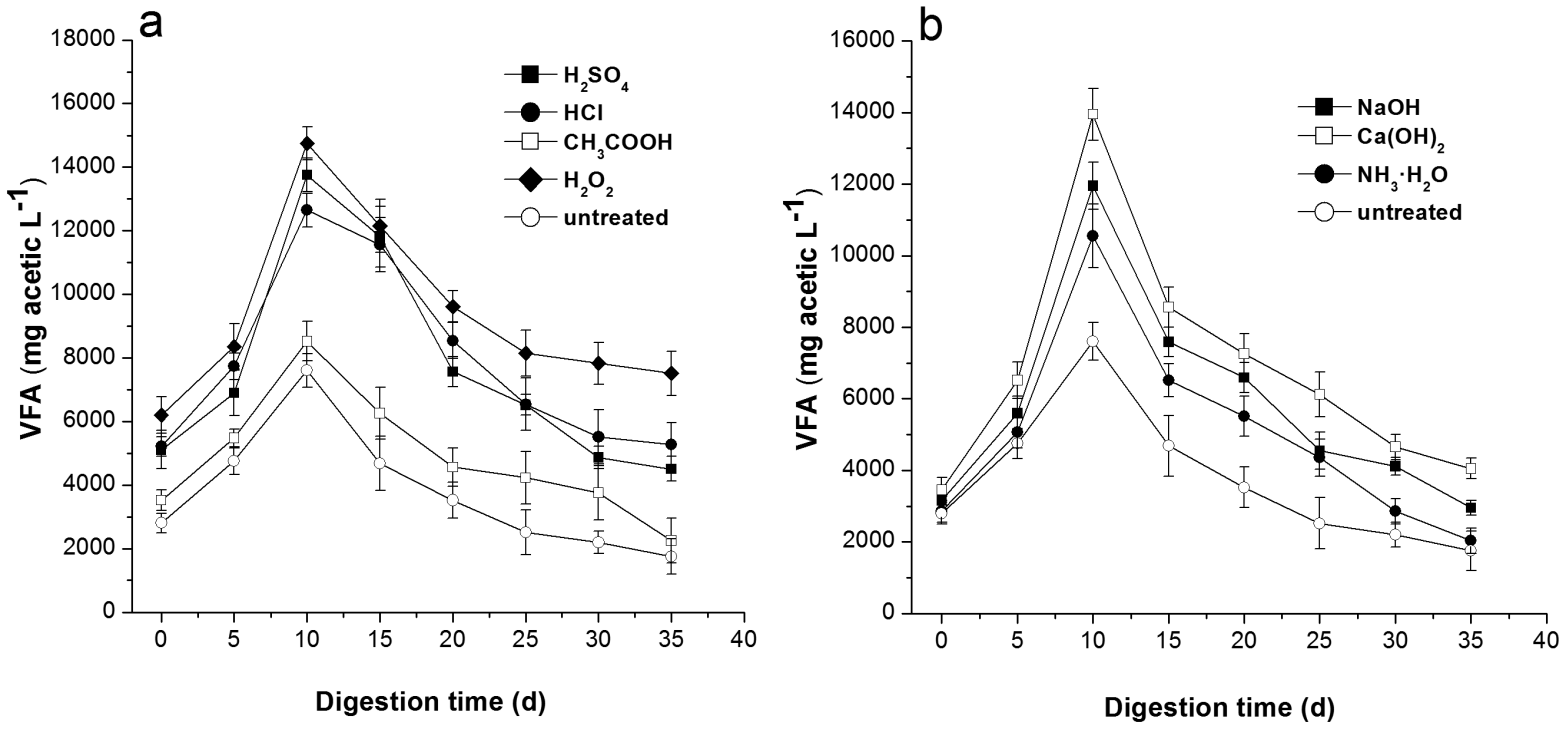
Change in the VFA of pretreated corn straw during digestion. (a) Acid pretreatment; (b) Alkaline pretreatment. Data was expressed at mean ± deviation of triplicate measurements.

The VFA concentration of each pretreatment initially increased (Fig. 4) and then decreased, which is contrary to the trend of the pH curve. The VFA content of the fermentation broth from the pretreated straw increased more sharply than that of the untreated corn straw. This result can be attributed to the significantly higher soluble substance content of the pretreated corn straw compared with the untreated samples. Among the seven pretreatments, the average VFA concentrations (mg acetic L^−1^) of the pretreatments during the AD were as follows: 7629 (H_2_SO_4_), 7879 (HCl), 4821 (CH_3_COOH), 9321 (H_2_O_2_), 5810(NaOH), 6818 (Ca(OH)_2_), and 4964 (NH_3_·H_2_O). The highest VFA values were observed for H_2_O_2_ in the acid treatment, whereas the lowest was observed for CH_3_COOH. This result is consistent with the results of the hemicellulose, cellulose, and lignin decomposition and methane yield (Table 1), which further confirmed the effectiveness of H_2_O_2_ in biodegrading the lignocellulosic structure of straws. Large amounts of hemicellulose and cellulose are converted into simple sugars, lipids (fats) into fatty acids, amino acids, and short-chain organic acids (butyric acid, propionic acid, acetate, and acetic acid), all of which are utilized by methanogens for methane production [15]. In the alkaline pretreatments, the highest VFA content was observed after using Ca(OH)_2_. This result was consistent with the observations from the methane yield experiments, but contradicted the lignocellulosic composition results where degradation of the lignin fraction was highest after NaOH pretreatments. This disparity can be explained by the fact that successful biogasification is not only affected by the sufficient soluble component available for the anaerobic bacteria but also by the balance between methanogens and acidogens [39]. The excessively high concentration of OH^−^ in NaOH likely inhibited acetogenesis and disturbed this balance. However, this hypothesis warrants further investigation.

### Economic Performance of the Pretreatment Methods

The effectiveness of a pretreatment is not only based on the effectiveness of AD but also on the economic performance. Table 3 compares the economic performance of the pretreatments at the optimal concentrations for methane yield. H_2_O_2_ and H_2_SO_4_ showed the lowest costs among the acid pretreatments. However, H_2_O_2_ was more favorable because it produced higher methane yields than H_2_SO_4_. In the alkaline pretreatments, although no great difference in the expenses was observed between the Ca(OH)_2_ and NaOH pretreatments, Ca(OH)_2_ produced is slightly advantageous over NaOH as it generates a higher methane yield. Therefore, with respect to economic performance and effectiveness, H_2_O_2_ and Ca(OH)_2_ can be considered as the most suitable pretreatments for corn straw.

**Table 3:** Economic performance of the different pretreatments.

	Chemicals	Concentration	Price a(CNY)	Cost ^b^(CNY)	Methane yield (mL CH_4_ gVS^−1^)
Acid	H_2_SO_4_	2%	21	2.57	175.6
	HCl	2%	15	4.92	163.4
	CH_3_COOH	4%	12.5	9.34	145.1
	H_2_O_2_	3%	6	3.6	216.7
Alkaline	NaOH	8%	9	4.2	163.5
	Ca(OH)_2_	8%	9.5	4.58	206.6
	NH_3_•H_2_O	10%	9	19.28	168.3

a The price was collected from the Sinophram Chemical Reagent Co. Ltd, Beijing China, and the unit of H_2_SO_4_, HCl, CH_3_COOH, H_2_O_2_, and NH_3_.H_2_O price was per 500 mL, NaOH and Ca(OH)_2_ was per 500 g. CNY is the abbreviation for Chinese Yuan, and a dollar is equivalent to 6.12 CNY on Oct 1, 2012; Bank of China. ^b^ The cost was calculated based on the pretreatment of 1 kg corn straw.

Recently, some researchers combined chemical and physical treatments to improve the biodegradability of lignocellulose composition. High temperature (120–250°C) is often used in combination with dilute acids or base in a pressure cell for much shorter durations. For instance, Saha et al. [40] found the 74% higher saccharification yield wheat straw was subjected to 0.75% v/v of H_2_SO_4_ at 121°C for 1 h. Cara et al. [41] shown that olive tree biomass pretreated with 1.4% H_2_SO_4_ at 210°C resulted in 76.5% of hydrolysis yields. Rocha et al. [42] reported that ethanol yield as high as 0.47 g/g glucose was achieved in fermentation tests with cashew apple bagasse pretreated with diluted H_2_SO_4_ at 121°C for 15 min. These studies showed the advantage of combination treatment on solubilizing the lignocellulosic composition and shortening the pretreatment time. Nevertheless, depending on the process temperature, some sugar degradation compounds such as furfural and aromatic lignin degradation compounds are detected, and affect the microorganism metabolism in the fermentation step [40]. Furthermore, the pretreatment of high temperature combined with chemicals consumes a substantial amount of energy, and need high facility investment and high treatment cost.

In the present study, although pretreatment time (7 day) was longer than that of chemical treatment with the addition of heat and pressure, the contents of hemicelluloses, cellulose, and lignin fractions of corn straw was greatly reduced, which was contribute to the enhancement of methane production. Furthermore, using single chemicals have no excessive energy consumption and less operation cost. Since cost reduction and low energy consumption are required for an effective pretreatment, chemical pretreatment without the addition of heat and pressure would be desirable to optimize the effectiveness on the process. As for the longer incubation time of the chemical pretreatment, more efforts should be made to investigate the combination of chemicals and low temperature (Below 100°C) pretreatment to shorten the incubation time and improve the anaerobic digestion efficiency.

## Conclusions

Four acid pretreatments (H_2_SO_4_, HCl, CH_3_COOH, and H_2_O_2_) and three alkaline pretreatments (NaOH, Ca(OH)_2_, and NH_3_·H_2_O) for improving the methane yield of corn straw were compared. All pretreatments were effective in the biodegradation of the lignocellulosic structure. Straw pretreated with 3% H_2_O_2_ and 8% Ca(OH)_2_ elicited the highest methane yields of 216.7 and 206.6 mL CH_4_ g VS^−1^, which are 115.4% and 105.3% higher than that of the untreated straw, respectively. H_2_O_2_ and Ca(OH)_2_ are economically and effectively superior to the other pretreatments. Therefore, H_2_O_2_ and Ca(OH)_2_ are both recommended as the pretreatments for improving the methane yield of straw.
