# Correction: Comparison of Seven Chemical Pretreatments of Corn Straw for Improving Methane Yield by Anaerobic Digestion

**DOI:** 10.1371/journal.pone.0101617

**Published:** 2014-06-27

**Authors:** The PLOS ONE Staff

The affiliation for the fourth author is incorrect. Zhiying Yan is not affiliated with #2 but with #1, Chengdu Institute of Biology, Chinese Academy of Science, Chengdu, Sichuan, PR China.
